# Hybrid kyphoplasty with short-versus intermediate- and long-segment pedicle screw fixations for the management of thoracolumbar burst fractures

**DOI:** 10.1186/s12891-024-07320-5

**Published:** 2024-03-07

**Authors:** Kuan-Nien Chou, Peng-Wei Wang, Ming-Hsuan Chung, Da-Tong Ju

**Affiliations:** Department of Neurological Surgery, Tri-Service General Hospital, National Defense Medical Center, 114 Taipei, Taiwan (R.O.C.)

**Keywords:** Posterior pedicle fixation, Cement augmentation, Burst fracture, Thoracolumbar spine

## Abstract

**Background:**

This study aimed to determine if the hybrid short-segment (HSS) technique is a good alternative to the intermediate-segment (IS) and long-segment (LS) techniques in pedicle screw fixations for acute thoracolumbar burst fractures (TLBFs).

**Methods:**

In this retrospective evaluation, we examined 43 patients who underwent surgical treatments, including one- or two-level suprajacent (U) and infrajacent (L) pedicle screw fixations, for acute single-level TLBFs with neurological deficits between the T11 and L2 levels from July 2013 to December 2019. Among these patients, 15 individuals underwent HSS (U1L1), 12 received IS (U2L1), and 16 underwent LS (U2L2) fixations. Supplemental kyphoplasty of the fractured vertebral bodies was performed exclusively in the HSS group. Our analysis focused on assessing blood loss and surgical duration. Additionally, we compared postoperative thoracolumbar kyphotic degeneration using the data on Cobb angles on lateral radiographic images acquired at three time points (preoperatively, postoperative day 1, and follow-up). The end of follow-up was defined as the most recent postoperative radiographic image or implant complication occurrence.

**Results:**

Blood loss and surgical duration were significantly lower in the HSS group than in the IS and LS groups. Additionally, the HSS group exhibited the lowest implant complication rate (2/15, 13.33%), followed by the LS (6/16, 37.5%) and IS (8/12, 66.7%) group. Implant complications occurred at a mean follow-up of 7.5 (range: 6–9), 9 (range: 5–23), and 7 (range: 1–21) months in the HSS, IS, and LS groups. Among these implant complications, revision surgeries were performed in two patients in the HSS group, two in the IS group, and one in the LS group. One patient treated by HSS with balloon kyphoplasty underwent reoperation because of symptomatic cement leakage.

**Conclusions:**

The HSS technique reduced intraoperative blood loss, surgical duration, and postoperative implant complications, indicating it is a good alternative to the IS and LS techniques for treating acute single-level TLBFs. This technique facilitates immediate kyphosis correction and successful maintenance of the corrected alignment within 1 year. Supplemental kyphoplasty with SpineJack® devices and high-viscosity bone cements for anterior reconstruction can potentially decrease the risk of cement leakage and related issues.

## Background

Traumatic burst fractures of the spine most commonly occur at the thoracolumbar level, where excessive axial loading forces act on the vulnerable biomechanical junction between the thoracic and lumbar spines [1]. The AO Spine Thoracolumbar Spine Injury Classification System labels vertebral burst fractures involving the posterior vertebral walls and one or both endplates as type A3 and A4 spinal injuries [2]. In burst fractures, fragments of bone spread in all directions, causing spinal deformity and disability [3]. Surgical treatment is usually recommended for thoracolumbar burst fractures (TLBFs) because of the risks of spinal deformity and/ or neurological deficits. However, the high complexity and variety of TLBFs has led to debate about the best surgical treatment strategy, including posterior fixation, anterior reconstruction, circumferential fusion, minimal vertebral cement augmentation, and hybrid approaches [4]. Anterior reconstruction techniques effectively provide anterior column support to improve implant failure and durable spinal deformity correction and achieve nerve decompression by resection and reconstruction of fracture vertebral fragments with posterior protrusion compared with posterior reconstruction techniques. However, the posterior approach has a lower risk of damage to internal organs and vascular structures, and it offers superior canal decompression and demands relatively low technical requirements in comparison to the anterior approach [5]. Moreover, meta-analysis studies have shown that the surgical duration is shorter and blood loss is lower with the posterior approach than with the anterior approach [6–8]. Thus, these advantages of the posterior approach have rendered it an appealing choice in appropriate cases of TLSBFs.

Various posterior fixation techniques with implants consisting of bilateral pedicle screw and rod fixations are used in either one or two levels adjacent to the fractured vertebra: short-segment (SS) fixation – one-level fixation cranial and one-level fixation caudal to the fractured vertebra (U1L1); intermediate-segment (IS) fixation – one-level fixation cranial and two-level fixation caudal (U1L2) or two-level fixation cranial and one-level fixation caudal to the fractured vertebra (U2L1); and long-segment (LS) fixation – two-level fixation cranial and two-level fixation caudal to the fractured vertebra (U2L2). Other posterior fixation techniques are also used, including SS pedicle fixation with additional pedicle screws on the fractured vertebrae and LS pedicle screw fixation with extension of more cranial and caudal levels. The decreased thoracolumbar spinal range of motion (ROM) in extension, lateral bending, and axial rotation have been shown in a finite element analysis of posterior pedicle screw fixations [9]. Among the various pedicle screw fixation techniques, the best preservation of physiological ROM was achieved by SS pedicle screw fixation, but it was accompanied by increased ROM over the fractured vertebra and von Mises stress on implants in flexion [9]. The highest rate (≤ 54%) of early implant failure and re-kyphosis in SS pedicle screw fixation results from the lack of anterior support of the fractured vertebra with dynamic instability in the TLBFs [10]. Compared with SS, LS pedicle screw fixation facilitates better correction of spinal alignment and a lower frequency of implant failures but with prolonged surgical duration and significantly increased blood loss [10, 11]. Similarly, thoracolumbar ROMs in flexion and extension were found in IS (U2L1) and SS pedicle screw fixations, but less von Mises stress and strain energy were observed on screws in IS fixations. Therefore, for single-level TLBFs, one finite element analysis suggested that U2L1 with IS pedicle screw fixation is better than SS and LS pedicle screw fixations [9].

Hybrid TLBF surgery was introduced in 2018 by Spiegl et al. In this method, SS pedicle screw fixation is supplemented with kyphoplasty of the fractured vertebral body to achieve better intraoperative and postoperative correction of spinal kyphosis and stability [12, 13]. Similar surgical techniques for treating TLBFs have been previously reported [14–16]. In finite element studies, the implant failure rate of SS pedicle screw fixation was reduced through additional cement augmentation of the fractured vertebra, which effectively reduced the amount of von Mises stress on the pedicle screws and rods and increased the stiffness of fractured vertebrae [17–19]. Compared with the anterior approach, the additional cement augmentation in the posterior approach increased the surgical safety and cost-effectiveness to achieve anterior support while avoiding the prolonged surgical duration, excessive implantations, and surgical complications associated with the anterior approach [20, 21]. Herein, we present an alternative technique—hybrid short-segment (HSS) pedicle screw fixation—for the treatment of TLBFs. This technique combines kyphoplasty with cement augmentation of the fractured vertebra and U1L1 short-segment pedicle screw fixation. We aimed to compare the clinical and radiographic outcomes of HSS pedicle screw fixation with IS (U2L1) and LS (U2L2) pedicle screw fixations for the treatment of unstable single-level type A3 or A4 TLBFs with neurological deficits.

## Methods

### Study design

**Fig. 1:**
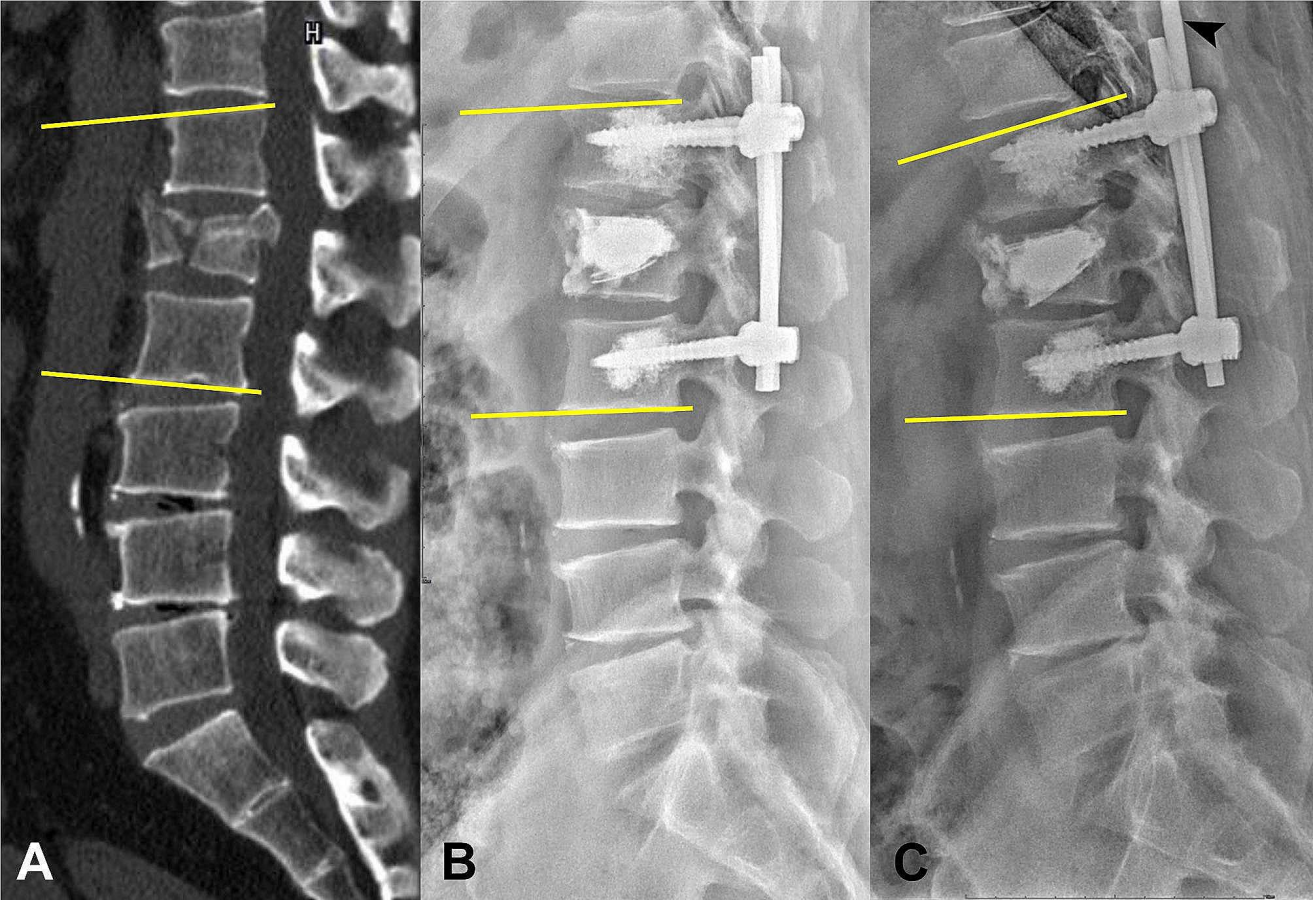
A 61-year-old male patient with an A4-type osteoporotic burst fracture at the L1 level and a McCormack load-sharing score of 8 underwent short-segment cement-augmented pedicle screw fixation with kyphoplasty and SpineJack® implantation. **A**: The Cobb angle of the thoracolumbar segment was measured on a sagittal spine computed tomography scan (angle_pre)_. **B**: Lateral spine radiography on postoperative day 1 showing angle_post_. **C**: Pedicle screw and rod dislodgement (arrowhead) at 9-month postoperative lateral spine radiography follow-up and angle_end_. angle_pre_, preoperative Cobb angle; angle_post_, 1-day postoperative Cobb angle; angle_end_, 9-month follow-up Cobb angle

This retrospective study was conducted at a general reference teaching hospital between July 2013 and December 2019. Patients with acute single-level traumatic TLBF with neurological deficits (T11–L2 level A3 or A4 injuries with retropulsed vertebral fragments) treated using a posterior decompression surgical approach and pedicle screw fixations were enrolled in this study. The surgical methods compared were HSS (U1L1), IS (U2L1), and LS (U2L2) pedicle screw fixations. The HSS group underwent either balloon kyphoplasty (BKP) or SpineJack^®^ (SJ; an expandable intravertebral implant, Stryker Corp., Kalamazoo, MI) kyphoplasty with additional four-screw U1L1 pedicle screw fixations. The American Society of Anesthesiology physical status (ASA-PS) scale was used for patient enrollment, with inclusion criteria limited to patients falling within ASA-PS class 1 and 2 [22]. This measure was implemented to mitigate potential bias stemming from comorbid conditions in the analysis of surgical technique utilization and associated risk of surgical complications. Additionally, patients with a history of malignancies, fractured vertebrae at more than one level, and spinal surgery; those without neurological deficits; and those who had been treated with other surgical techniques or had not undergone regular follow-up imaging (≥ 6 months) were excluded. A series of thoracolumbar spinal images obtained via computed tomography (CT), bone mineral density (BMD) scans, and preoperative lateral radiography were conducted at preoperative, postoperative day 1, and postoperative end-stage time points. The postoperative end-stage was defined as the most recent postoperative radiographic image or implant complication occurrence. The severity of each TLBF was evaluated on preoperative CT scans using the McCormack load-sharing classification [23], including the characteristic parameters for vertebral comminuted fractures, fragment displacement, and kyphosis correction (Table 1). McCormack et al. performed anterior reconstruction with posterior fixation on burst fractures, with fractures scored ≥ 7 on the McCormack load-sharing classification being classified as severe [23]. The American Spinal Injury Association Impairment Scale was used to evaluate preoperative and postoperative neurological function. Data on segmental Cobb angles (between the superior endplate of the vertebra above and inferior endplate of the vertebra below the fractured vertebra) were recorded from lateral radiographic images (Fig. 1) [24]. Imaging data were analyzed to determine the therapeutic effects of the three different surgical techniques on kyphosis. Additionally, for comparing the three approaches, we evaluated surgical risks in terms of the intraoperative blood loss volume and surgical duration.

**Table 1:** McCormack load sharing classification (Reference [23])

Score	1 point	2 points	3 points
Comminution	< 30%	30–60%	> 60%
Fragment displacement	Minimal displacement	Displacement < 50%	Displacement > 50%
Kyphosis correction	3°	4°–9°	≥10°

### Imaging assessment

The characteristics observed in all preoperative CT imaging studies were compatible with those of acute type A3 or A4 burst fractures. BMD was evaluated using T-scores from dual-energy X-ray absorptiometry of the lumbar segment (L3–5) or femoral neck. Osteoporosis was defined by T-scores of ≤ − 2.5. The severity and type of vertebral fractures were evaluated by preoperative spinal CT and scored using the McCormack load-sharing classification. For each patient, the Cobb method was used to measure the angle of the thoracolumbar segment on serial supine lateral spine radiographic or sagittal spine CT scan images obtained at three time points (preoperatively [angle_pre_], postoperative day 1 [angle_post_], and postoperative end-stage [angle_end_]) (Fig. 1). To correct the intrinsic error in Cobb angle measurement, all radiographic images obtained at the three different time points for each patient were assessed twice by the second author using the same protractors. All data were presented after arithmetic calculations.

### Surgical procedures

HSS pedicle screw fixation was performed on patients in the prone position on a Jackson table under general anesthesia. After identifying the fractured vertebra, the spine was exposed by making a posterior midline incision and performing a bilateral decompressive laminectomy, with preservation of the spinal process and interconnecting ligaments. A unilateral or bilateral transpedicular approach was used to insert working cannulas into the fractured vertebra, which is not recommended for pedicles with comminuted fractures. Reaming tools were used to create a working space between the anterior and posterior vertebral bodies and between the endplate and anterior two-thirds of the vertebral body, where appropriate balloons or SJs were implanted. The balloons were inflated slowly until an adequate vertebral height was achieved. Similarly, the SJs were expanded to restore vertebral height. Subsequently, poly(methyl methacrylate) (PMMA) bone cement was injected gradually via the working cannulas until impending cement extravasation was observed under fluoroscopic guidance. Four pedicle screws were inserted into the pedicles at one level cranial and caudal according to each surgeon’s chosen technique and anatomical landmarks, and two rods with appropriate curves were connected.

IS and LS pedicle screw fixations were performed using a posterior midline incision followed by decompressive laminectomy. In the IS group, four transpedicular screws were inserted into the two vertebrae cranial to the fractured vertebra and two transpedicular screws into one vertebra caudal to the fractured vertebra. In the LS group, eight transpedicular screws were inserted into the pedicles of the two vertebrae cranial and caudal to the fractured vertebra. In both the IS and LS groups, two rods appropriate to the instrumentation level were used. After an intraoperative evaluation of spinal stability, the rods were bound to each other in some cases using transverse connectors at a minimum of two levels.

The ReBorn Essence Lumbar Fixation System (New Taipei City, Baui Biotech, Co., Ltd., Taiwan) was used in the three patient groups. Paramedian incisions were performed in patients treated with percutaneous screws. Cement-augmented pedicle screws were used in patients with osteoporosis. A C-shaped intraoperative fluoroscopic device was used to perform all three procedures.

### Statistical analyses

SPSS for Windows statistical software version 21.0 (IBM SPSS Statistics for Windows, IBM Corp., Armonk, NY, USA) was used to assess and reassess the demographic and radiographic parameters of the different surgical techniques for the treatment of TLBFs. One-way ANOVA was used to compare the demographic characteristics of the patients in each group (Table 2). A *p*-value of < 0.05 was considered statistically significant. The Scheffe post hoc test was performed to compare between-group differences after a significant one-way ANOVA result. The same statistical analyses were performed to compare implant complication rates and Cobb angle data in the three surgical groups at different time points (Table 3). The Mann–Whitney *U* test was used to compare the surgical duration and blood loss of the patients treated with percutaneous or pedicle screws in the HSS group (Table 4). The Shapiro–Wilk test was used to assess the normality of the data.

**Table 2:** Demographic characteristic of the patients

Characteristics	HSS group	IS group	LS group	*p*-value	Post-hoc test
	(*n* = 15)	(*n* = 12)	(*n* = 16)		
Age	49.47 ± 19.35	48.83 ± 12.31	52.56 ± 15.97	0.805	
Sex				0.979	
Male	8	6	8		
Female	7	6	8		
Level				0.339	
T11	0	0	0		
T12	2	4	5		
L1	11	5	6		
L2	2	3	5		
Osteoporosis	3	2	3	0.976	
Load sharing score				0.384	
4	2	0	0		
5	0	1	1		
6	1	5	5		
7	4	2	4		
8	6	2	5		
9	2	2	1		
Load sharing score				0.257	
≤ 6	3	6	6		
≥ 7	12	6	10		
Neurological status(preoperative)				0.775	
A	0	0	1		
B	0	1	0		
C	3	1	2		
D	12	10	13		
Neurological status(postoperative)				0.386	
B	0	0	1		
C	0	1	0		
D	5	1	4		
E	10	10	11		
Surgical duration	159.93 ± 56.06	237.5 ± 79.92	301.56 ± 80.42	< 0.001	HSS < IS, LS
Volume of blood loss (mL)	219.00 ± 232.73	331.25 ± 237.41	684.38 ± 483.35	0.008	LS > IS, HSS
Follow-up (months)	11.93 ± 17.63	10.00 ± 5.13	11.75 ± 10.74	0.912	

*Abbreviation* HSS, hybrid short-segment; IS, intermediate-segment; LS, long-segment

**Table 3:** Comparison among the three types of surgery

Variables	HSS group	IS group	LS group	*p*-value	Post-hoc test
	(*n* = 15)	(*n* = 12)	(*n* = 16)		
**Cobb angle**					
Preoperative	18.90 ± 6.16	19.52 ± 9.39	20.64 ± 7.15	0.808	
Postoperative day 1	7.53 ± 7.66	14.49 ± 7.24	13.91 ± 6.61	0.022	NS
End stage of postoperative	10.15 ± 6.80	22.15 ± 7.28	19.54 ± 7.53	< 0.001	HSS < IS, LS
Immediate surgical correction	11.37 ± 6.31	5.03 ± 5.22	6.74 ± 6.71	0.028	HSS > IS
Delayed correction effect	8.75 ± 4.65	−2.63 ± 6.48	1.11 ± 7.15	< 0.001	HSS > IS, LS
Postoperative loss of correction at end stage	-2.61 ± 3.75	-7.66 ± 3.46	-5.63 ± 4.70	0.009	HSS < IS
**Implant complications**				0.018	HSS < IS
Yes	2	8	6		
No	13	4	10		

*Abbreviation* HSS, hybrid short-segment; IS, intermediate-segment; LS, long-segment; NS, not significant

**Table 4:** Comparison between percutaneous screw and pedicle screw (HSS group)

Variables	Percutaneous screw	Pedicle screw	*p*-value
	(*n* = 11)	(*n* = 4)	
Operative time	159.82 ± 64.29	160.25 ± 29.85	0.851
Volume of blood loss (mL)	229.55 ± 227.71	190.00 ± 280.00	0.177

## Results

### Patient characteristics

In this study, there were 43 patients (22 men and 21 women; mean age: 50.4 [range: 24–87] years) with single-level TLBFs, including 15, 12, and 16 who underwent HSS (6 with SJ and 9 with BKP), IS, and LS pedicle screw fixations, respectively. No significant sex and age differences were observed among the three groups. The majority of fractures were on the T12 and L1 levels. The severity of TLBFs and preoperative neurological function were comparable between the three groups. No patient suffered neurological deterioration due to the surgical treatment. The end-stage imaging data were observed at a mean follow-up of 11.2 months. The HSS group had a lower mean blood loss volume and shorter mean surgical duration than the IS and LS groups (Table 2). Only one patient in the IS group and one in the LS group were treated with posterior percutaneous pedicle screw fixation (NOVA Minimally Invasive System; New Taipei City, Baui Biotech, Co., Ltd., Taiwan). In the HSS group, 11 patients underwent paramedian incisions with percutaneous screws, and no significant differences were observed in surgical duration and blood loss between patients treated with percutaneous screws or pedicle screws (Table 4).

### Effects on kyphosis

To compare the immediate and long-term effects of the three techniques on kyphosis correction, we recorded and analyzed the Cobb angle of each patient obtained at three time points (preoperatively [angle_pre_], postoperative day 1 [angle_post_], and postoperative end-stage [angle_end_]) (Table 3). For each patient, we identified the immediate effects of their surgery on kyphosis by calculating the angle_pre_ minus angle_post_ (representing the immediate surgical correction). To evaluate the maintenance of the corrected alignment, we calculated the angle_pre_ minus angle_end_ (representing the delayed correction effect). To identify progressive kyphosis after surgery, we calculated the angle_post_ minus angle_end_ (representing end-stage postoperative loss of correction).

The average Cobb angle was significantly lower in the HSS group than in the other groups on postoperative day 1 (*p* = 0.022, one-way ANOVA); however, marginally significant differences were observed among the three groups in Scheffe’s post hoc test (HSS < IS, *p* = 0.054; HSS < LS, *p* = 0.058). This may have been due to the limited number of patients in each group. Another Scheffe’s post hoc test showed significantly lower end-stage postoperative Cobb angles in the HSS group than in the LS and IS groups. Moreover, compared with IS pedicle screw fixation, HSS showed a better immediate corrective effect and less progression of postoperative kyphosis. The HSS pedicle screw fixation technique produced the best final correction effect and maintained the corrected alignment at a mean follow-up of approximately 1 year.

### Surgical complications of kyphosis correction

**Fig. 2:**
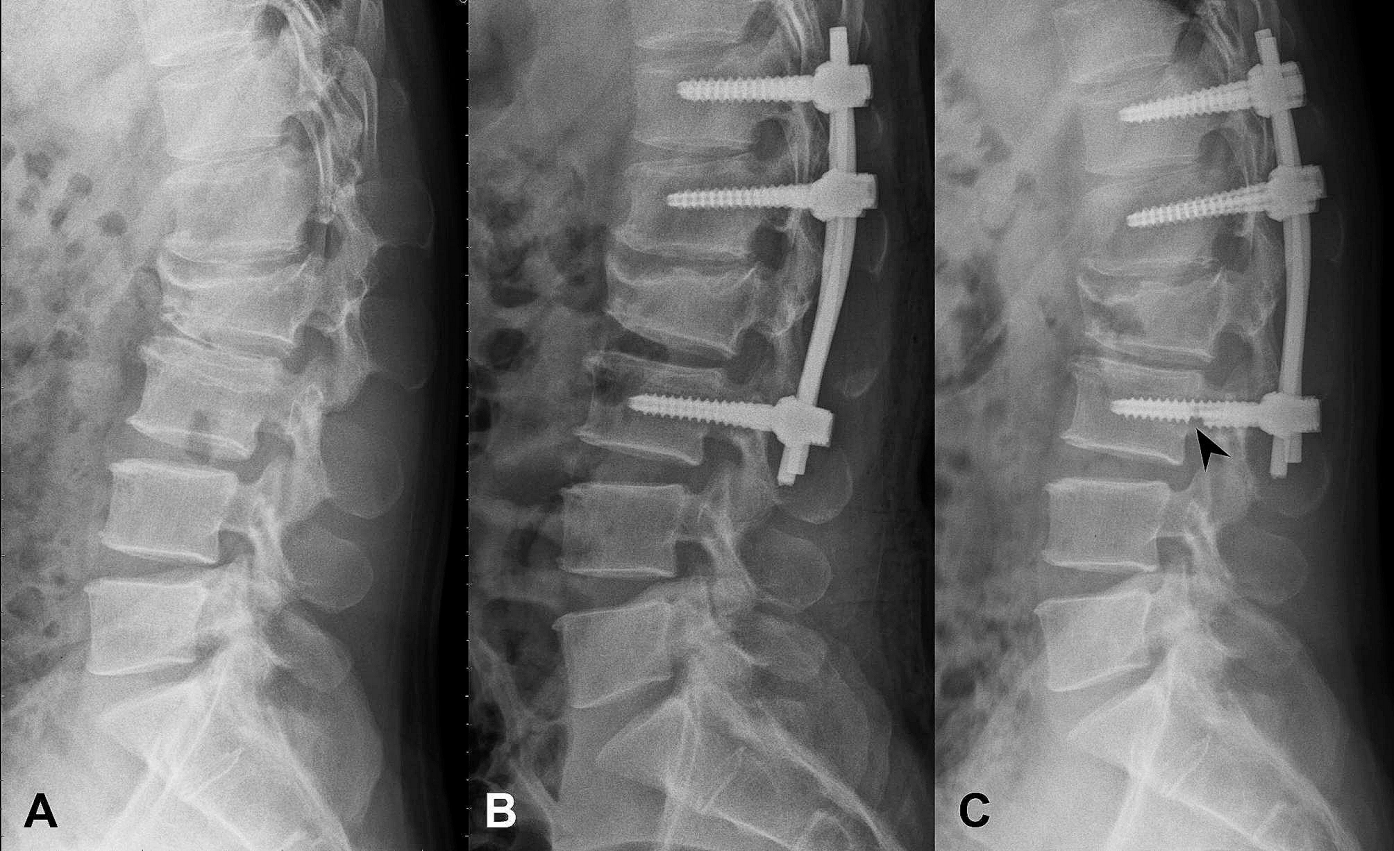
A 58-year-old female patient with an A3-type burst fracture at the L2 level and a McCormack load-sharing score of 5 underwent intermediate-segment pedicle screw fixation. **A**: Preoperative lateral spine radiography. **B**: Postoperative lateral spine radiography on day 1. **C**: A broken pedicle screw (arrowhead) seen in lateral spine radiography at the 6-month postoperative follow-up

**Fig. 3:**
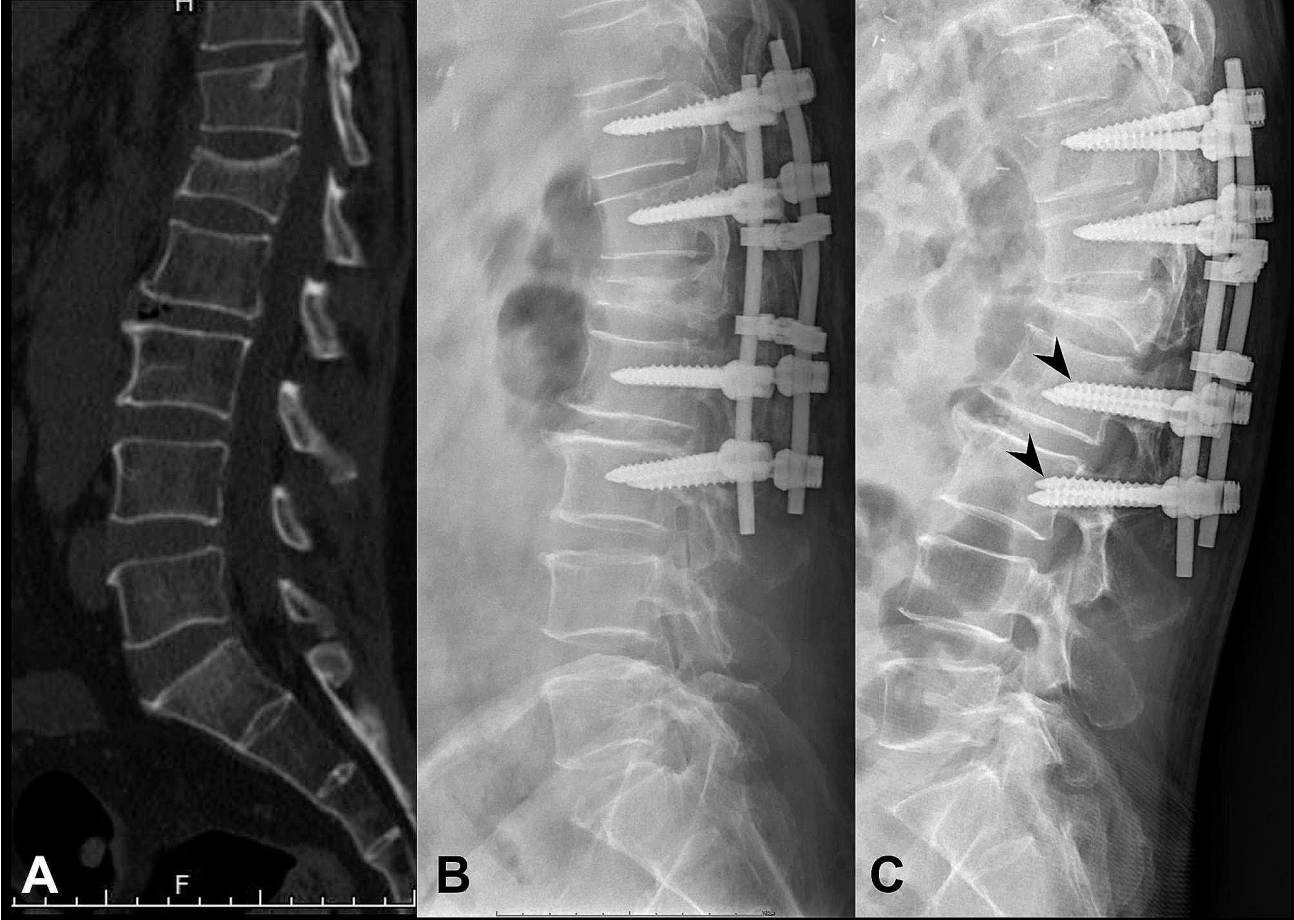
A 62-year-old male patient with an A3-type burst fracture at the L1 level and a McCormack load-sharing score of 5 underwent long-segment pedicle screw fixation. **A**: Preoperative lateral spine computed tomography scan. **B**: Postoperative lateral spine radiography on day 1. **C**: Inferior pedicle screw pullout (arrowhead) seen in lateral spine radiography at the 1-month postoperative follow-up

The implant complications observed in this study were dislodgement of the pedicle screw and rod (Fig. 1C), pedicle screw breakage (Fig. 2C), and pedicle screw pullout (Fig. 3C). Two HSS patients (2/15; 13.3%) were found to have pedicle screw and rod dislodgement at 6- and 9-month postoperative follow-ups, respectively (Fig. 1). Both patients required revision surgery. In the IS group, eight patients (8/12; 66.7%) experienced implant complications at a mean follow-up of 9 (range: 5–23) months. Among these patients, two received revision surgeries. Five of these patients (5/12; 41.6%) presented with inferior pedicle screw pullout, two (2/12; 16.7%) with pedicle screw and rod dislodgement, and one (1/12; 8.3%) with inferior pedicle screw breakage (Fig. 2). In the LS group, six patients (6/16; 37.5%) suffered implant complications at a mean follow-up of 7 (range: 1–21) months. Among them, one patient underwent revision surgery. One patient (1/16; 6.3%) presented with pedicle screw and rod dislodgement and five (5/16; 31.2%) with pedicle screw pullout (one on a superior level and four on inferior levels, Fig. 3). One patient with inferior pedicle screw pullout in the IS group and one in the LS group had medical histories of osteoporosis. Among the three groups, the HSS group had the lowest implant complication rate. Implant complications were found in the three groups within the first postoperative year, mainly at a mean of 9 months in the IS group and 7 months in the LS group. The IS group had the shortest follow-up (mean: 10.0 months) because eight patients (8/12; 66.7%) experienced implant complications at a mean follow-up of 9 (range: 5–23) months.

## Discussion

Although orthosis is typically recommended as the initial treatment for relatively less severe injuries, such as A3 TLBF, particularly in patients below 60 years of age without neurological deficits [25, 26], we included patients with acute neurological deficit in both A3 and A4 TLBFs treated with posterior decompression and fixation in this study. Previous studies have evaluated implant complications and long-term kyphosis correction outcomes after treatment of TLBFs with SS (U1S1) and LS (U2L2) pedicle screw fixations [10, 11]. The advantages of posterior SS pedicle screw fixation include a small incision wound, short surgical duration, and early mobilization resulting from less involvement of lumbar motion segments [11]. . However, a high implant complication rate in SS pedicle screw fixations has been widely reported, with an incidence of ≤ 54% [10]. This finding may be attributed to the untreated vertebral instability and poor resistance to the anterior compressive force in SS pedicle screw fixations without further support [9, 27–29] and has been improved by additional pedicle screws or cement augmentation on fractured vertebrae. The use of supplemental cement augmentation or additional pedicle screws on fractured vertebrae has been reported to effectively reduce the amount of von Mises stress on pedicle screws in the unfractured vertebra and rods, thereby improving implant failures in SS pedicle screw fixation [19, 30]. The addition of one or two more pedicle screws at the fractured vertebrae has been found to present the similar postoperative spinal stability and to lower the rates of progressive kyphosis and implant failure in SS pedicle screw fixations [31, 32]. Zhang et al. found that supplemental BKP improved vertebral instability to produce better clinical and radiological outcomes compared with the use of additional pedicle screws on fractured vertebrae in SS pedicle screw fixations [33]. Additionally, Cho et al. found that TLBF treatment with combined SS pedicle screw fixation and PMMA vertebroplasty provided immediate vertebral stability and prevented any incidence of posterior instrumentation failure at 2-year follow-ups [34]. Finite element studies have also reported a reduction in the implant failure rate of SS pedicle screw fixation through additional cement augmentation of the fractured vertebra, which effectively reduced the amount of von Mises stress on the implants and increased the stiffness and stability of fractured vertebrae [17–19]. The injected PMMA bone cement effectively provides anterior column support; however, there is a concern regarding its potential to interrupt bone healing in fractured vertebrae, especially in patients with normal bone mineral density. The injury to intraosseous blood vessels as occurrence of burst fractures compromise the blood supply of the vertebral body bone tissue and hinder bone healing, which appears to be the most likely cause of its progression to non-union and osteonecrosis [35]. Untreated vertebral instability and incomplete bone healing of fractured vertebrae in TLBFs can lead to inadequate anterior column support and kyphotic degeneration following posterior pedicle screw fixation treatment. Additionally, these issues may also result in back pain after the removal of implants [36, 37]. The placement of bone graft within kyphoplasty devices in fractured vertebrae has been reported to achieve immediate anterior column reconstruction and long-term vertebral bone strength by promoting bone healing [38]. However, the potential resorption or necrosis of the bone graft could adversely affect the bone healing process [39, 40]. Despite these considerations, compared to bone grafts, PMMA bone cement achieves superior filling of the bone defect and stabilization of the vertebral body interior. Herein, we performed BKP or SJ kyphoplasty with PMMA bone cement to correct spinal kyphosis and achieve vertebral stability. The corrected spinal kyphosis and resulting vertebral stability reduce the amount of von Mises stress on implants, thereby supporting our result that the HSS technique had the lowest implant complication rate (13.3%). Among the patients in the HSS group, a delayed postoperative vertebral collapse was noted in a 61-year-old male patient with an A4-type burst fracture at the L1 level treated with SJ kyphoplasty and low-viscosity PMMA bone cement (Fig. 1). This may have resulted from a dislodged pedicle screw and rod. Furthermore, a 21-year-old male patient with TLBFs at the L1 level was treated with BKP, low-viscosity PMMA bone cement, and SS pedicle screw fixation. The patient presented with right-sided allodynia in the dermatome below the L2 level and grade 4 weakness (Medical Research Council scale) in knee extension and ankle dorsiflexion due to cement leakage [41]. These symptoms were compatible to cement leakage with subsequent right-sided lateral recess stenosis on the L1–2 level. The revision surgery was performed to remove extravasated cement.

Kyphoplasty, which is distinct from vertebroplasty, facilitates the low-pressure injection of PMMA bone cement, thereby minimizing the incidence of cement leakage in cement augmentation procedures designed to address acute TLBF [42–44]. Kyphoplasty also produces better immediate postoperative kyphosis correction and less progressive kyphotic degeneration than vertebroplasty and nonsurgical treatments [45]. The high incidence of cortical defects and instability of crushed vertebrae in TLBFs has been attributed to bone cement leakage [46, 47]. Improvement in the components of conventional high-viscosity PMMA bone cement is necessary to prevent cement leakage [48]. Additionally, a greater amount of PMMA bone cement injection is associated with a higher risk of cement leakage [42]. In kyphosis treatment, the use of SJs showed less risk of cement leakage than BKP because the lower amount of injected PMMA bone cement achieved better intraoperative and durable kyphosis correction in traumatic vertebral fractures [49–51]. It’s crucial to emphasize that SJs differ from space-occupying devices like in BKP. Instead, SJs are expandable implants designed to provide direct support to the vertebral body through vertical expansion and prevent the potential protrusion of fractured bone fragments during expansion procedures, setting them apart from the ballooning procedures involved in BKP [52]. The use of SJs with high-viscosity bone cement for anterior reconstruction in the HSS treatment of TLBF appeared to achieve better vertebral correction and reduced risk of cement leakage, especially in those with greater severity on the McCormack load-sharing classification.

IS and LS pedicle screw fixations are used to lengthen the arm level of the implants, and both can improve spinal stability in the treatment of TLBFs. Moreover, they have lower implant complication rates than SS pedicle screw fixations [53, 54]. Similar von Mises stress and strain energies were found on implants of IS and LS pedicle screw fixations in flexion and extension. Compared with SS pedicle screw fixation, IS and LS pedicle screw fixations reduced von Mises stress and strain energy on pedicle screws and may have contributed to lower implant complication rates [9]. SS and IS with caudal one-level pedicle screw fixations had similar thoracolumbar physiological ROM preservation in extension, lateral bending, and axial rotation. However, the ROM in flexion was increased by approximately 50% in the IS and SS groups versus 26.5% in the LS group [9]. In comparison with the caudal two-level screwing, posterior fixation with caudal one-level pedicle screws has also been reported to show less resistance to the compressive force acting anteriorly [55]. These findings support our finding that the IS technique had the highest implant complication rate (66.7%), mainly with inferior pedicle screw pullout and dislodgement.

Few studies have assessed and compared the surgical risks, implant complication rate, and clinical efficacy of HSS, IS, and LS pedicle screw fixations in the treatment of TLBFs. Tan et al. found no difference in kyphotic degeneration between the combined anterior and posterior approach and the posterior approach, but a significantly longer surgical duration and more intraoperative blood loss were observed in the combined approach [56]. In our study, HSS pedicle screw fixation required shorter surgical durations and resulted in less intraoperative blood loss than IS and LS pedicle screw fixations. Previous meta-analyses found no significant differences in implant-related complications between SS and LS pedicle screw fixations or between the combined anterior and posterior approach and the posterior approach [10, 56]. However, in the present study, the HSS group had the lowest implant complication rate (13.33%), followed by the LS (37.5%) and IS (66.7%) groups. Aly found no significant differences in postoperative kyphosis correction and progressive kyphosis outcomes between SS and LS groups [10]. In our study, HSS pedicle screw fixation produced better immediate kyphosis correction outcomes and maintained the corrected alignment with the least progressive kyphosis at a mean follow-up of 1 year.

This study had several limitations. First, patients with a history of malignancy, spinal surgery, and vertebral fractures of more than one level were excluded from the study. We only compared three different surgical techniques in this study; therefore, the HSS technique was not compared with other surgical techniques, such as U1L2 posterior and SS pedicle screw fixations with additional index level screws on the fractured vertebrae. Hence, our sample was relatively small, consisting of a total 43 patients (15 in HSS, 12 in IS and 16 in LS). This limited sample size reduces the validity and reliability of our results and prevents more specific comparisons between subsets. Additionally, most patients in all three groups had suffered mild neurological deficits of grade D on the American Spinal Injury Association Impairment Scale. Hence, we could not statistically evaluate the relationship between the severity of neurological deficits and surgical blood loss and duration. Second, some neurosurgeons found the HSS technique preferable in the treatment of single-level TLBFs, and patients were assigned to one of the three surgical techniques based on patient evaluations and neurosurgeon preference rather than sequential selection. Considering the presence of concurrent medical conditions in elderly patients and the restricted spinal mobility following posterior pedicle screw fixations in young patients, the utilization of the LS technique was less common in our hospital. These considerations introduced potential bias in our study, particularly given the wide age range of patients, spanning from 24 to 87 years old. Thus, sampling could not be randomized because of the retrospective study design. Significant differences in surgical decisions, blood loss volume, and surgical durations occurred among the 13 neurosurgeons in our hospital because of differences in their surgical techniques, experience, and aptitudes, all of which may have introduced further bias. Third, strict standing lateral thoracolumbar radiographic examinations were not performed, which led to a significant bias in the measurement of Cobb angles at different stages. Further studies should address these limitations by conducting more detailed preoperative assessment of medical conditions and postoperative functional outcome evaluations, such as the use of the visual analog scale and Oswestry disability index. While our findings may not be universally applicable to TLBFs, they nonetheless serve as a valuable foundation for designing future studies that can address the unique and challenging circumstances presented by patient cohorts with longer follow-up periods.

## Conclusions

HSS pedicle screw fixation is recommended as a favorable alternative to the IS and LS techniques for treating acute single-level TLBFs, with significantly less operative blood loss, fewer implant complications, and shorter surgical duration. Moreover, this procedure facilitates immediate kyphosis correction and successful maintenance of the corrected alignment within 1 year. The use of SJ kyphoplasty with an appropriate volume of high-viscosity PMMA bone cement for anterior reconstruction can potentially decrease the risk of cement leakage and related issues.

## Data Availability

The data that support the results of this study are available on request from the first author, KN Chou, and are not publicly available owing to ethical restrictions.

## Abbreviations

TLBF: thoracolumbar burst fracture
SS: short segment
U1L1: one-level fixation cranial and one-level fixation caudal to the fractured vertebra
U1L2: one-level fixation cranial and two-level fixation caudal to the fractured vertebra
U2L1: two-level fixation cranial and one-level fixation caudal to the fractured vertebra
U2L2: two-level fixation cranial and two-level fixation caudal to the fractured vertebra
LS: long segment
HSS: hybrid short segment
IS: intermediate segment
ROM: range of motion
BKP: balloon kyphoplasty
CT: computed tomography
BMD: bone mineral density
SJ: SpineJack®
PMMA: poly(methyl methacrylate)
